# Echocardiographic functional determinants of survival in heart failure with abnormal ejection fraction

**DOI:** 10.3389/fcvm.2023.1290366

**Published:** 2023-11-21

**Authors:** Quirino Ciampi, Lauro Cortigiani, Nicola Gaibazzi, Fausto Rigo, Angela Zagatina, Karina Wierzbowska-Drabik, Jaroslaw D. Kasprzak, Ana Djordjevic-Dikic, Maciej Haberka, Andrea Barbieri, Ylenia Bartolacelli, Mauro Pepi, Scipione Carerj, Bruno Villari, Patricia A. Pellikka, Eugenio Picano

**Affiliations:** ^1^ Cardiology Department, Fatebenefratelli Hospital of Benevento, Benevento, Italy; ^2^ Cardiology Department, San Luca Hospital, Lucca, Italy; ^3^ Cardiology Department, Villa Salus Foundation/IRCCS San Camillo Hospital, Venice, Italy; ^4^ Cardiology Department, University of Parma, Parma, Italy; ^5^ Cardiology Department, Research Cardiology Center “Medika”, Saint Petersburg, Russian Federation; ^6^ Department of Internal Disease and Clinical Pharmacology, Medical University, Lodz, Poland; ^7^ Department of Cardiology, Medical University of Lodz, BieganskiSpecialty Hospital, Lodz, Poland; ^8^ Clinical Center of Serbia and School of Medicine, University of Belgrade, Cardiology Clinic, Belgrade, Serbia; ^9^ Cardiology Department, University of Silesia, Katowice, Poland; ^10^ Cardiology Division, Department of Biomedical, Metabolic and Neural Sciences, University of Modena and Reggio Emilia, Policlinico di Modena, Modena, Italy; ^11^ Paediatric Cardiology and Adult Congenital Heart Disease Unit, S. Orsola-Malpighi Hospital, Bologna, Italy; ^12^ Cardiology Division, Centro Cardiologico Monzino, IRCCS, Milano, Italy; ^13^ Cardiology Division, University Hospital Polyclinic G.Martino, University of Messina, Messina, Italy; ^14^ Department of Cardiovascular Medicine, Mayo Clinic, Rochester, MN, United States; ^15^ Biomedicine Department, CNR, Institute of Clinical Physiology, Pisa, Italy

**Keywords:** travel, publication, infrastructural funding from società italiana di ecocardiografia e cardiovascular imaging (SIECVI), stress echo, heart failiure, coronary flow reserve, contractile reserve, dilated cardiaomypothy

## Abstract

**Background and Aims:**

Patients with heart failure (HF) with reduced left ventricular (LV) ejection fraction (EF) have a heterogeneous prognosis, and assessment of coronary physiology with coronary flow velocity (CFV) and coronary flow velocity reserve (CFVR) may complement established predictors based on wall motion and EF.

**Methods and results:**

In a prospective multicenter study design, we enrolled 1,408 HF patients (age 66 ± 12 years, 1,035 men), with EF <50%, 743 (53%) with coronary artery disease, and 665 (47%) with normal coronary arteries. Recruitment (years 2004–2022) involved 8 accredited laboratories, with inter-observer variability <10% for CFV measurement. Baseline CFV (abnormal value >31 cm/s) was obtained by pulsed-wave Doppler in mid-distal LAD. CFVR (abnormal value ≤2.0) was assessed with exercise (*n* = 99), dobutamine (*n* = 100), and vasodilator stress (dipyridamole in 1,149, adenosine in 60). Inducible myocardial ischemia was identified with wall motion score index (WMSI) stress > rest (cut-off Δ ≥ 0.12). LV contractile reserve (CR) was identified with WMSI stress < rest (cutoff Δ ≥ 0.25). Test response ranged from score 0 (EF > 30%, CFV ≥ 32 cm/s, CFVR > 2.0, LVCR present, ischemia absent) to score 5 (all steps abnormal). All-cause death was the only endpoint. Results. During a median follow-up of 990 days, 253 patients died. Independent predictors of death were EF (HR: 0.956, 95% CI: 0.943–0.968, *p* < 0.0001), CFV (HR: 2.407, 95% CI: 1.871–3.096, *p* < 0.001), CFVR (HR: 3.908, 95% CI: 2.903–5.260, *p* < 0.001), stress-induced ischemia (HR: 2.223, 95% CI: 1.642–3.009, *p* < 0.001), and LVCR (HR: 0.524, 95% CI: 0.324–.647, *p *= 0.008). The annual mortality rate was lowest (1.2%) in patients with a score of 0 (*n *= 61) and highest (31.9%) in patients with a score of 5 (*n* = 15, *p *< 0.001).

**Conclusion:**

High resting CFV is associated with worse survival in ischemic and nonischemic HF with reduced EF. The value is independent and additive to resting EF, CFVR, LVCR, and inducible ischemia.

## Introduction

Heart failure with reduced ejection fraction (HFrEF) is a clinical syndrome with heterogeneous etiology, characterized by signs or symptoms of heart failure (HF) with a left ventricular (LV) ejection fraction (EF) ≤ 40% (1). Patients with heart failure and mildly reduced EF (HFmrEF) show an LV EF between 41% and 49% and may benefit from similar therapies as in HFrEF (1, 2). Chronic HF with either reduced or mildly reduced EF remains a diagnostic challenge with many possible phenotypes determining the outcome. These distinct high-risk phenotypes are the lack of residual LV contractile reserve (CR), regional wall motion abnormality (RWMA) of ischemic (coronary) or non-ischemic (myocardial) origin, and the impaired functional status of coronary microcirculation (3). Stress echocardiography (SE) has a recognized role, with a class of recommendation 2b (“may be useful”) in chronic HF. The evaluation of RWMA recommended by guidelines can be enriched to include the simultaneous assessment of resting coronary flow velocity (CFV) and coronary flow velocity reserve (CFVR) in the left anterior descending (LAD) coronary artery (4–6). An altered coronary blood flow physiology is an early marker of HF, contributes to the progression of the disease, and is also a potentially actionable therapeutic target (7). The current study hypothesis was that a combination of resting and stress assessment of RWMA and CFV might identify different endotypes, with heterogeneous levels of risk, in chronic HF with reduced/mildly reduced EF. In this hypothesis-driven analysis of prospectively acquired data from accredited laboratories contributing to multicentre international studies (8), we assessed the prognostic contribution of the combined evaluation of CFV and RWMA in patients with HF, of ischemic and non-ischemic etiology, with reduced/mildly reduced EF.

## Methods

### Patients

**Figure 1:**
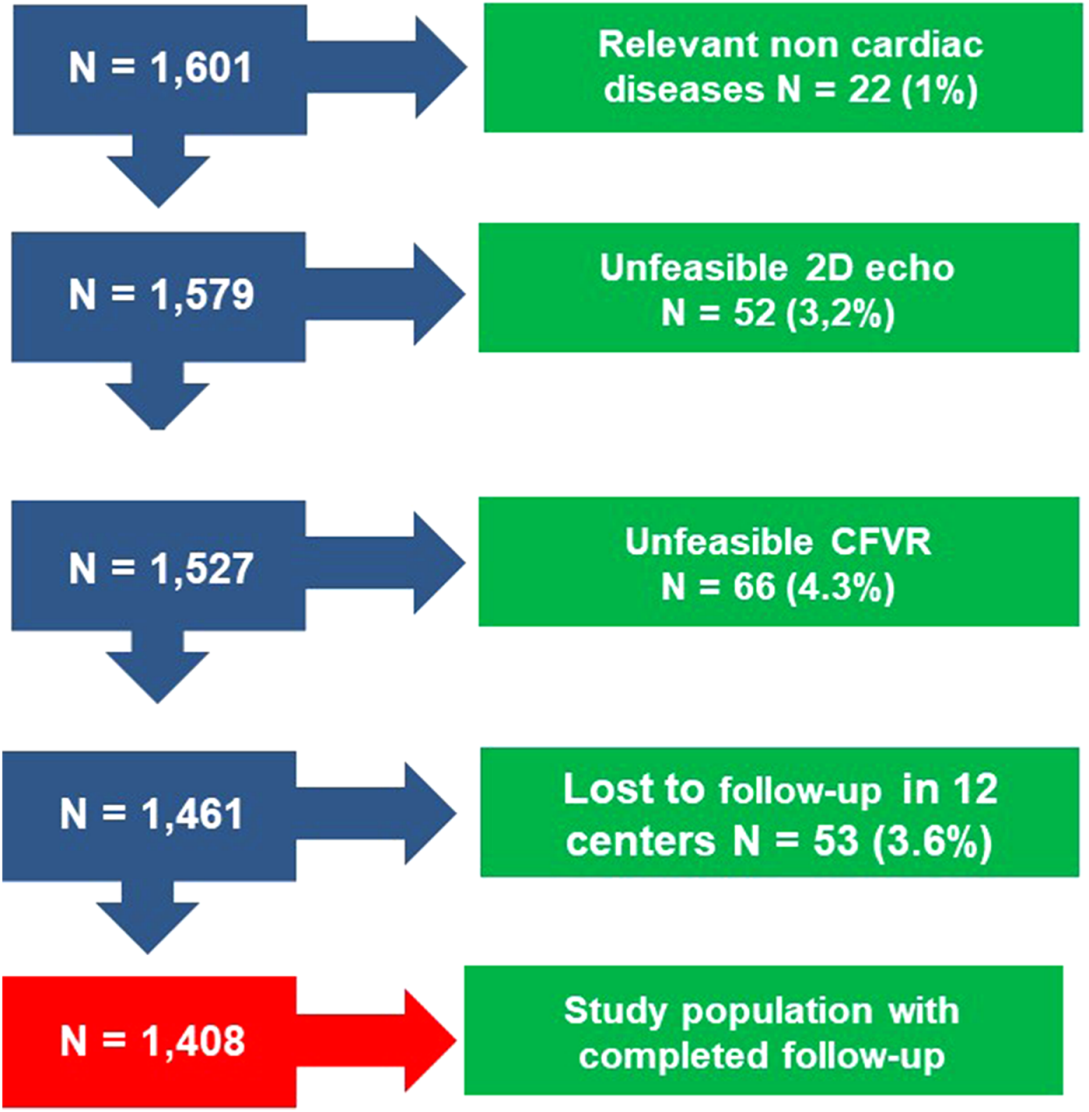
Consort diagram flow diagram showing how many individuals were excluded at each exclusion step.

The initial population comprised 1,601 patients prospectively enrolled at 8 cardiology institutions (Benevento, Lucca, Venice, Parma, Saint Petersburg, Lodz, Belgrade, Katowice) from 4 countries, in a study on CFVR started in 2002 and integrated into the SE 2030 from March 2021 onwards (8). Indication for SE was the assessment of myocardial viability and inducible ischemia in patients with HFrEF or HFmrEF. Exclusion criteria were clinically significant valvular or congenital heart disease, and prognostically relevant non-cardiac diseases (advanced cancer, end-stage renal disease, or severe obstructive pulmonary disease). All patients underwent transthoracic echocardiography (TTE) including resting CFV and SE with an assessment of CFVR of mid-distal LAD. The employed stress was semi-supine exercise (*n* = 99), high-dose dobutamine (*n* = 100), or high-dose vasodilator (*n* = 1,209; dipyridamole in 1,149, adenosine in 60), based on the patient's characteristics and laboratory expertise. Of 1,601 patients initially considered and present in the data bank, 66 (4.3%) were excluded from analysis for inadequate acoustic window precluding satisfactory imaging of LAD flow Doppler (for CFV and CFVR assessment), 52 (3.2%) for inadequate acoustic window precluding satisfactory imaging of endocardial borders, and 53 (3.6%) for missing follow-up data. Accordingly, 1,408 (1,035 [73%] men; mean [ ± SD] age 65 ± 11 years) with interpretable CFV and CFVR data, and complete follow-up data formed the study group (Figure 1). The study protocol was reviewed and approved by the institutional ethics committees, in its latest versions as a part of the more comprehensive SE 2020 study (148-Comitato Etico Lazio-1, July 16, 2016; Clinical trials.Gov Identifier NCT 030.49995) and stress echo 2030 study 291/294/295 Comitato Etico Lazio-1, March 8, 2021; Clinical trials.Gov Identifier NCT NCT050.81115) (8).

### TTE

TTE was performed using commercially available ultrasound machines equipped with multifrequency phased-array sector scan probes and with second harmonic technology. All patients underwent comprehensive TTE at rest. All measurements were taken by certified cardiologists according to the recommendations of the American Society of Echocardiography and the European Association of Cardiovascular Imaging (9).

To measure EF, LV end-diastolic volume, and end-systolic volume were assessed using various methods based on the available imaging views. Apical four- and two-chamber views were used primarily, and the biplane Simpson method was employed for measurement. In cases where biplane apical views were unavailable, a single-plane 4-chamber view area-length method was utilized. In situations where neither of these views was feasible, for non-distorted LVs, a linear measurement was obtained from the parasternal view using a linear method. The reduction in EF was categorized as follows: mild (41%–49%), moderate (30%–40%), or severe (<30%) LV dysfunction.

Electrocardiogram and blood pressure were monitored continuously. Criteria for interrupting the test were severe chest pain, diagnostic ST-segment shift, excessive blood pressure increase (systolic blood pressure ≥240 mmHg, diastolic blood pressure ≥120 mmHg), limiting dyspnea, maximal predicted heart rate, significant arrhythmias or limiting side effects. Wall motion score index (WMSI) was calculated in each patient at baseline and peak stress, in a four-point score ranging from 1 (normal) to 4 (dyskinetic) in a 17-segment model of the LV (10).

Coronary flow in the mid-distal segment of the LAD was visualized from the low parasternal long-axis view and/or modified apical two-, three-, or four-chamber views, using color Doppler flow mapping as a guide. Vendor-specific settings were pre-adjusted to optimize coronary flow imaging, and the color flow velocity was set to a range of 20–30 cm/s, with wall motion filters turned off. The sample volume was approximately 5 mm. All studies were digitally stored to facilitate offline review and measurements. For each time point, three optimal profiles of peak diastolic Doppler flow velocities were measured, and the results were then averaged.

Pulsed-Doppler assessment of rest CFV and stress CFVR was defined as the ratio between hyperemic peak and basal peak diastolic coronary flow velocities in mid-distal LAD. The procedure of acquisition was standardized between centers through a web-based learning module before starting data collection. All readers (one for each center) underwent quality control as previously described with <10% variability for CFV measurements (10).

### SE positivity criteria

The criteria of abnormal response were either determined *a priori* on the basis of previously established cutoff validated vs. prognostic endpoints (for EF, CFVR, WMSI, LVCR, and EF) or with a receiver-operating characteristics (ROC) analysis on the present data (for resting CFV). CFVR was considered abnormal when ≤2.0 (10). Severely abnormal EF was identified with resting EF < 30%. Myocardial ischemia was identified with inducible RWMA and WMSI stress > rest (cut-off Δ ≥ 0.12), corresponding to the worsening of 1 grade in at least 2 of 17 segments, or the worsening of 2 grades in 1 segment. LV contractile reserve (CR) was identified with WMSI stress < rest (cutoff Δ ≥ 0.25), corresponding to the improvement of 1 grade in at least 5 of 17 segments with a resting score ≥2. TTE + SE response was also expressed in a binary (normal/abnormal) fashion for each main variable, and the composite score in each patient ranged from score 0 (all steps normal) to score 5 (all steps abnormal).

### Follow-up data

Deaths were identified from the national health service database. Non-deceased participants were contacted directly. Mortality was the only endpoint. To avoid misclassification of the cause of death, overall mortality was considered. Follow-up was not censored at the time of coronary artery revascularization.

### Statistical analysis

Continuous variables are expressed as mean ± SD. Event rates were estimated with reverse Kaplan-Meier curves and compared by the log-rank test. Univariable analyses by Cox proportional hazards models were performed to assess the association between each candidate variable and outcome. Non-proportionality of the hazard was assessed using the Schoenfeld test.

To evaluate the ability of CFV to classify risk, we plotted ROC curves for the overall mortality included in the analysis. The C statistic, a measure of the area under the ROC curve, was calculated. Calculations of sensitivity, specificity, and accuracy were performed according to standard definitions.

The primary endpoint was the time-to-event analysis by a multivariable Cox proportional hazards model. Hazard ratios (HR) with the corresponding 95% confidence interval (CI) were estimated. The selection of independent predictors was performed for the logistic and proportional hazards model with a backward approach using a *p*-value of 0.10 as a threshold for inclusion in the model. Clinical variables and the sequential steps of rest EF, resting CFV, LVCR, inducible ischemia, and CFVR were sequentially included in the model and the global chi-square was calculated after each step. A significant increase after the addition of further variables indicated incremental prognostic value. All analyses were two-sided. Statistical significance was set at *p* < 0.05. All statistical calculations were performed using SPSS for Windows, release 20.0 (Chicago, Illinois).

## Results

The main clinical and echocardiographic features in study patients are reported in Table 1, with the overall population, divided into ischemic or non-ischemic etiology, according to previous history of myocardial infarction, coronary angiographic findings and coronary artery revascularizations.

**Table 1:** Clinical characteristics of the patients and main rest and stress findings.

	All patients (1,408 pts)	Ischemic HF (743 pts)	Non-ischemic HF (665 pts)	*p*-value
Age (years)	65 ± 11	67 ± 10	65 ± 14	*<0*.*001*
Sex, M/F, *N* (%)	1,035 (73%)	592 (80%)	443 (67%)	*<0*.*001*
	373 (37%)	151 (20%)	222 (33%)	
Type of stress:				
Exercise *N* (%)	99 (7.0%)	13 (2.0%)	86 (11.6%)	*<0* *.* *001*
Vasodilator *N* (%)	1,209 (85.9%)	605 (91.0%)	604 (81.3%)	
Dobutamine *N* (%)	100 (7.1%)	47 (7.0%)	53 (7.1%)	
Hypertensive patients *N* (%)	945 (67%)	550 (74%)	395 (59%)	*<0*.*001*
Diabetic patients *N* (%)	480 (34%)	277 (37%)	203 (30%)	*0*.*008*
LBBB *N* (%)	369 (26%)	93 (13%)	276 (42%)	*<0*.*001*
Previous myocardial infarction, *N* (%)	608 (43%)	608 (81%)	0	* *
Previous PCI/CABG, *N* (%)	639 (45%)	639 (86%)	0	* *
Beta blockers, *N* (%)	836 (59.4%)	439 (59.1%)	397 (59.7%)	*0*.*815*
Rest SBP (mmHg)	130 ± 19	130 ± 19	130 ± 20	*0*.*718*
Rest DBP (mmHg)	77 ± 12	78 ± 11	76 ± 13	*0*.*020*
Rest HR (bpm)	70 ± 12	67 ± 11	72 ± 12	*<0*.*001*
Stress HR (bpm)	92 ± 20	93 ± 22	90 ± 10	*0*.*052*
Rest WMSI	1.67 ± 0.45	1.64 ± 0.41	1.70 ± 0.49	*<0*.*001*
Stress WMSI	1.64 ± 0.46	1.67 ± 0.44	1.62 ± 0.47	*<0*.*001*
*Δ*WMSI (stress-rest)	−0.03 ± 0.24	0.02 ± 0.23	−0.08 ± 0.24	*<0*.*001*
LVCR, *N* (%)	169 (12%)	49 (7%)	120 (18%)	*<0*.*001*
Stress-induced ischemia, *N* (%)	224 (17%)	168 (23%)	76 (11%)	*<0*.*001*
Rest LV EF (%)	39 ± 17	41 ± 6	38 ± 8	*<0*.*001*
Rest CVF of LAD (cm/s)	33.5 ± 15.4	32.2 ± 14.3	35.1 ± 16.8	*<0*.*001*
High CFV, *N* (%)	662 (47%)	312 (42%)	350 (53%)	*<0*.*001*
CFVR of LAD	2.02 ± 0.53	2.00 ± 0.56	2.05 ± 0.51	*0*.*089*
Reduced CFVR, *N* (%)	725 (51%)	338 (52%)	337 (51%)	*0*.*563*

LBBB, left bundle branch block; PCI, percutaneous coronary intervention; CABG, coronary artery by-pass grafting; SBP, systolic blood pressure; DBP, diastolic blood pressure; HR, heart rate, bpm: beats per minute; WMSI, wall motion score index; LVCR, left ventricular contractile reserve; EF, ejection fraction; CVF, coronary flow velocity; LAD, left anterior descending coronary artery; CFVR, coronary flow velocity reserve.

### Resting TTE findings

The resting echocardiographic LV EF in the entire study group was 39 ± 17%. Resting CFV was 33.5 ± 15.4 cm/s, with higher values in non-ischemic compared to ischemic etiology (Table 1).

### SE findings

No major complications occurred during the test. Abnormal CFVR value of was ≤2 was found in 725 (51%) patients. Average value of CFVR was 2.02 ± 0.53, with lower values in patients with HFrEF compared to HFmrEF (1.93 ± 0.56 and 2.08 ± 0.51, respectively, *p* < 0.001], and similar values both in ischemic or non-ischemic etiology (Table 1). LVCR was more prevalent in patients without compared to those with diabetes mellitus [145 (15.6%) of 928 vs. 24 (5.0%) of 480, respectively, *p* < 0.001]; in non-ischemic compared to ischemic etiology [120 (18.0%) of 665 vs. 49 (6.6%) of 743, respectively, *p* < 0.001], and in HFrEF compared to HFmrEF [99 (18.3%) of 541 vs. 69 (8.0%) of 867, respectively, *p* < 0.001]. Inducible RWMA was more prevalent in patients with compared to those without diabetes mellitus [116 (24.2%) of 480 vs. 128 (13.8%) of 928, respectively, *p* < 0.001], in ischemic compared to non-ischemic etiology [168 (22.6%) of 743 vs. 76 (11.4%) of 665, respectively, *p* < 0.001)], and similar in HFrEF compared to HFmrEF [85 (15.7%) of 541 vs. 159 (18.3%) of 867, respectively, *p* = 0.205]. CFVR was correlated with rest-stress change in WMSI (*r* = −0.323, *p* < 0.001). The average score TTE + SE score was 2.18 ± 1.13, similar in ischemic vs. non-ischemic HF (2.17 ± 1.10 vs. 2.19 ± 1.17, *p* = 0.703).

**Figure 2:**
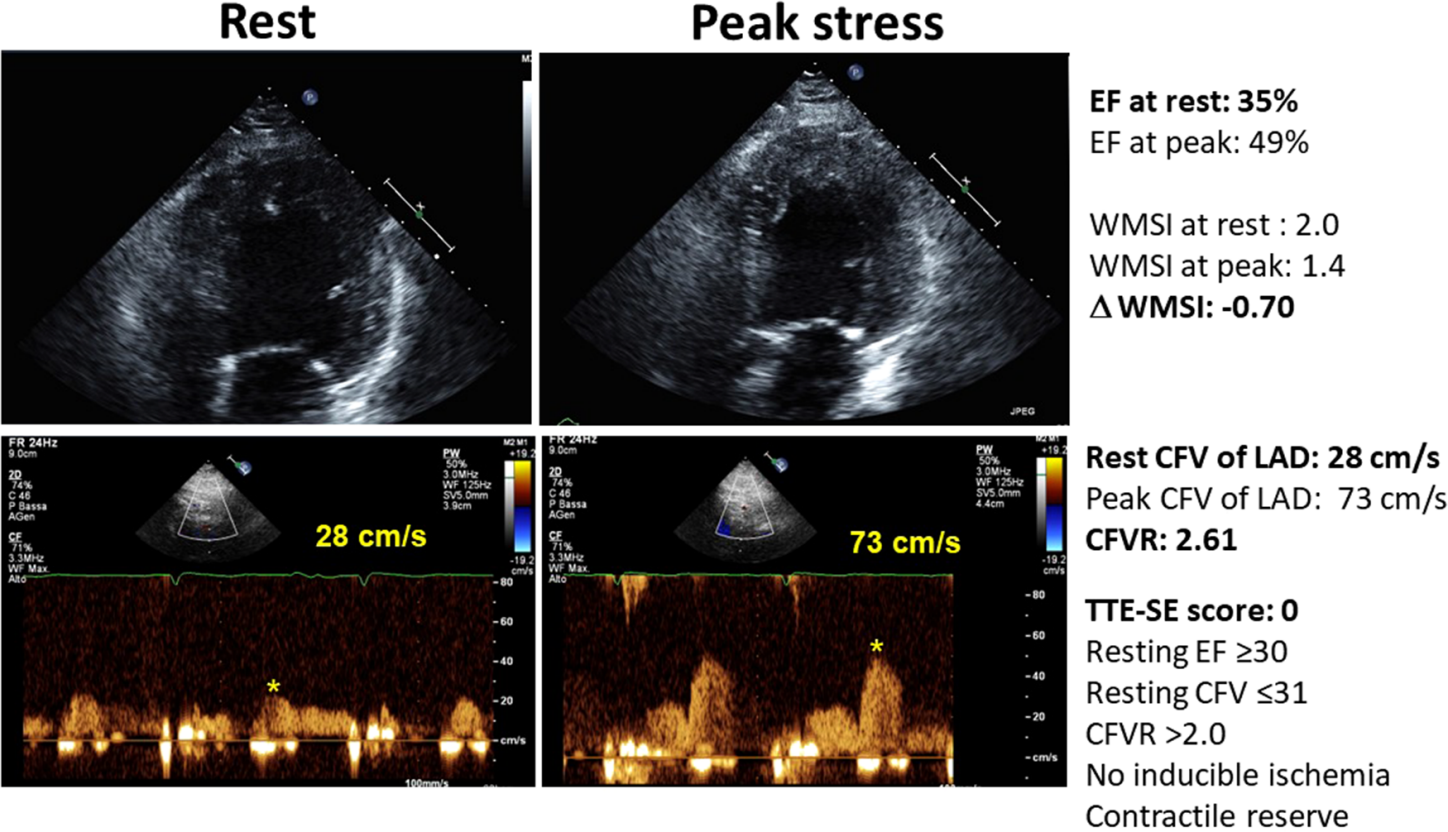
The normal pattern of normal resting and peak flow velocity with dipyridamole, with normal reduction of LV end-systolic volumes and regional wall motion improvement during stress. Upper panel, color-Doppler signal; middle panel, pulsed-wave Doppler signal; lower panel: end-systolic frame of the LV.

**Figure 3:**
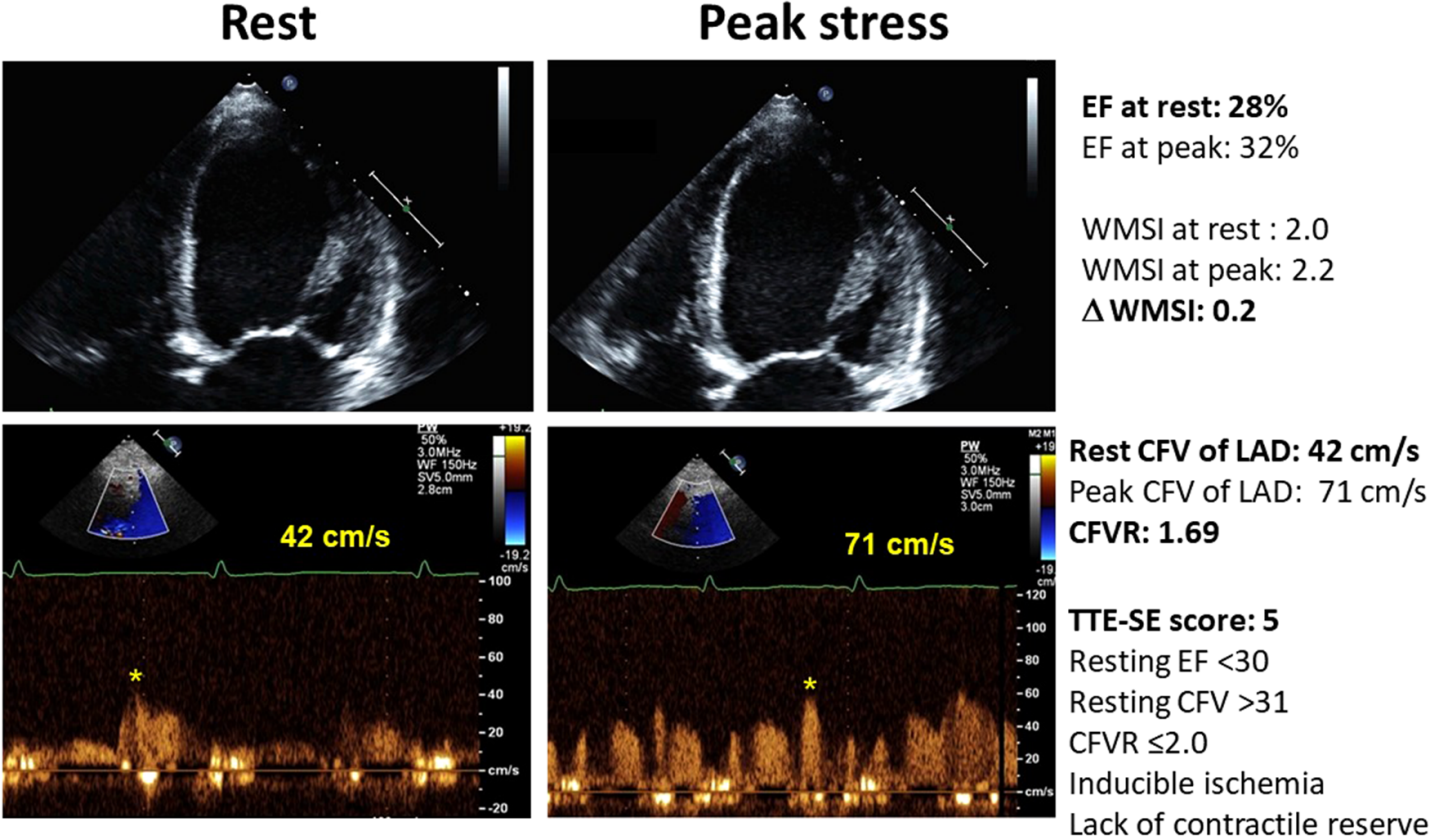
The abnormal pattern of high resting flow velocity with reduced CFVR with dipyridamole stress, with unchanged LV end-systolic volumes and no regional wall motion improvement during stress. Upper panel, color-Doppler signal; lower panel, pulsed-wave Doppler signal.

Figure 2 shows an example of a patient with a normal CFVR, with normal resting CFV, the presence of LVCR, and no inducible RWMA. Figure 3 shows an example of a patient with an abnormally increased resting CFV, with reduced CFVR, absence of LVCR, and inducible RWMA.

### Follow-up events

During a median follow-up of 990 days (1st quartile 384, 3rd quartile 2,238 days), there were 253 (18.0%) deaths.

### Outcome prediction

Resting CFV ≥ 32 cm/s was considered abnormal according to a receiver-operating characteristics analysis (sensitivity 58.1%, specificity 55.5%, the area under ROC curve 0.583, 95% CI: 0.556–0.609). Mortality at 5 years was lowest (10%) in patients in the lowest quintile (≤24 cm/s) and highest (50%) in patients in the highest (≥40 cm/s) quintile of CFV The annualized mortality rate progressively increased with increasing quintiles of CFV, from 2% in the lowest up to 5% for the highest quintile.

An increased CFV was a significant predictor of increased risk when a separate analysis was run for patients with CAD (*n* = 743, 136, 18.3% deaths) or non-CAD (*n* = 665, 117, 17.6% deaths). In CAD cohort, CFV > 32 cm/s was associated with an increased risk at univariable [HR 95% CI: 2.850 (2.025–4.011), *p* < 0.001] and multivariable analysis [HR 95% CI: 2.005 (1.394–2.884), *p* < 0.001]. In no CAD cohort, CFV ≥ 32 cm/s was also associated with an increased risk at univariable [HR 95% CI 1.932 (1.364–2.897), *p* < 0.001] and multivariable analysis [HR 95% CI 1.488 (1.1.014–2.186), *p* = 0.043].

**Figure 4:**
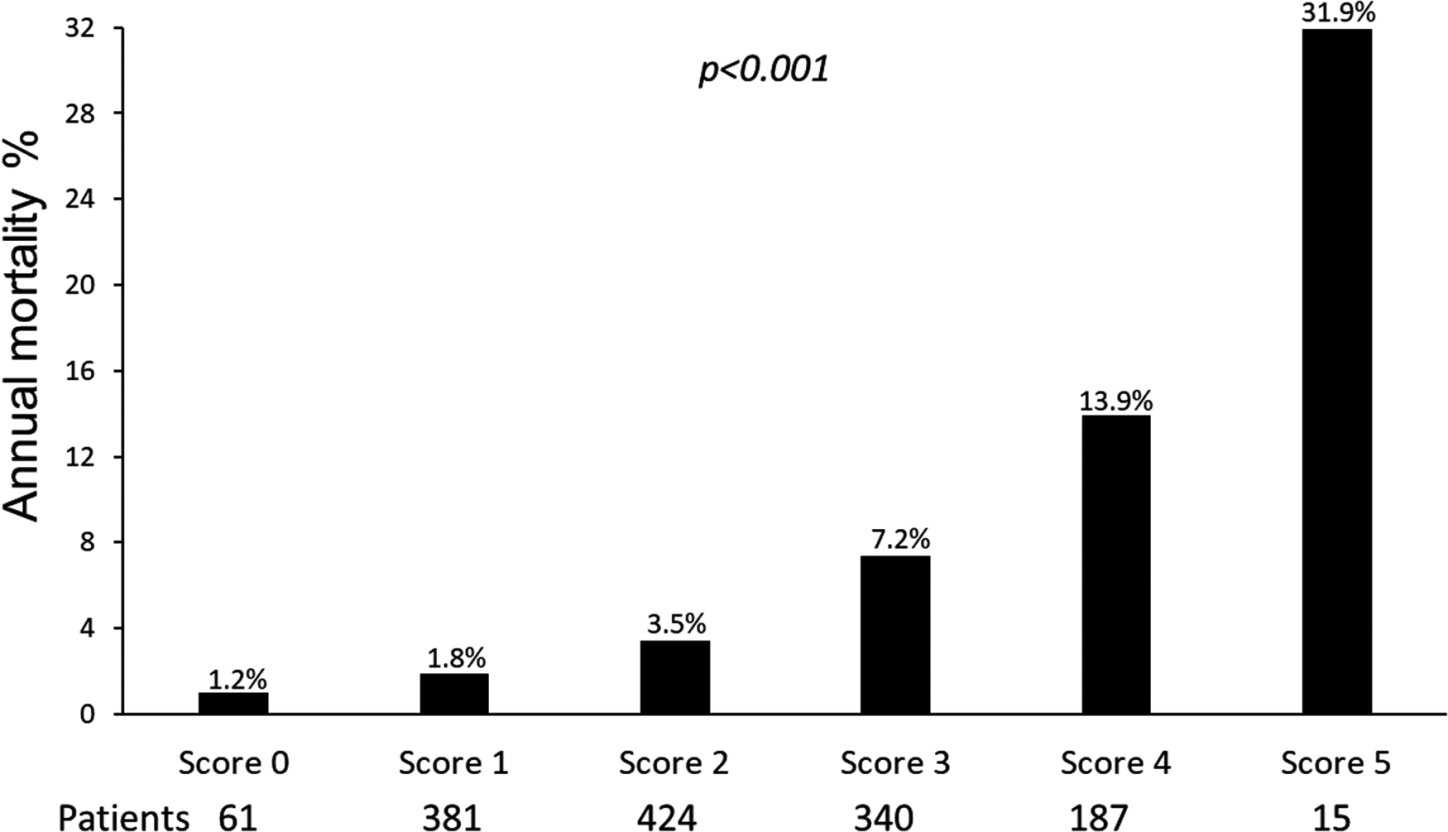
Annual mortality rate according to the TTE-SE score values, ranging from 0 (normal) to 5 (severely abnormal).

**Figure 5:**
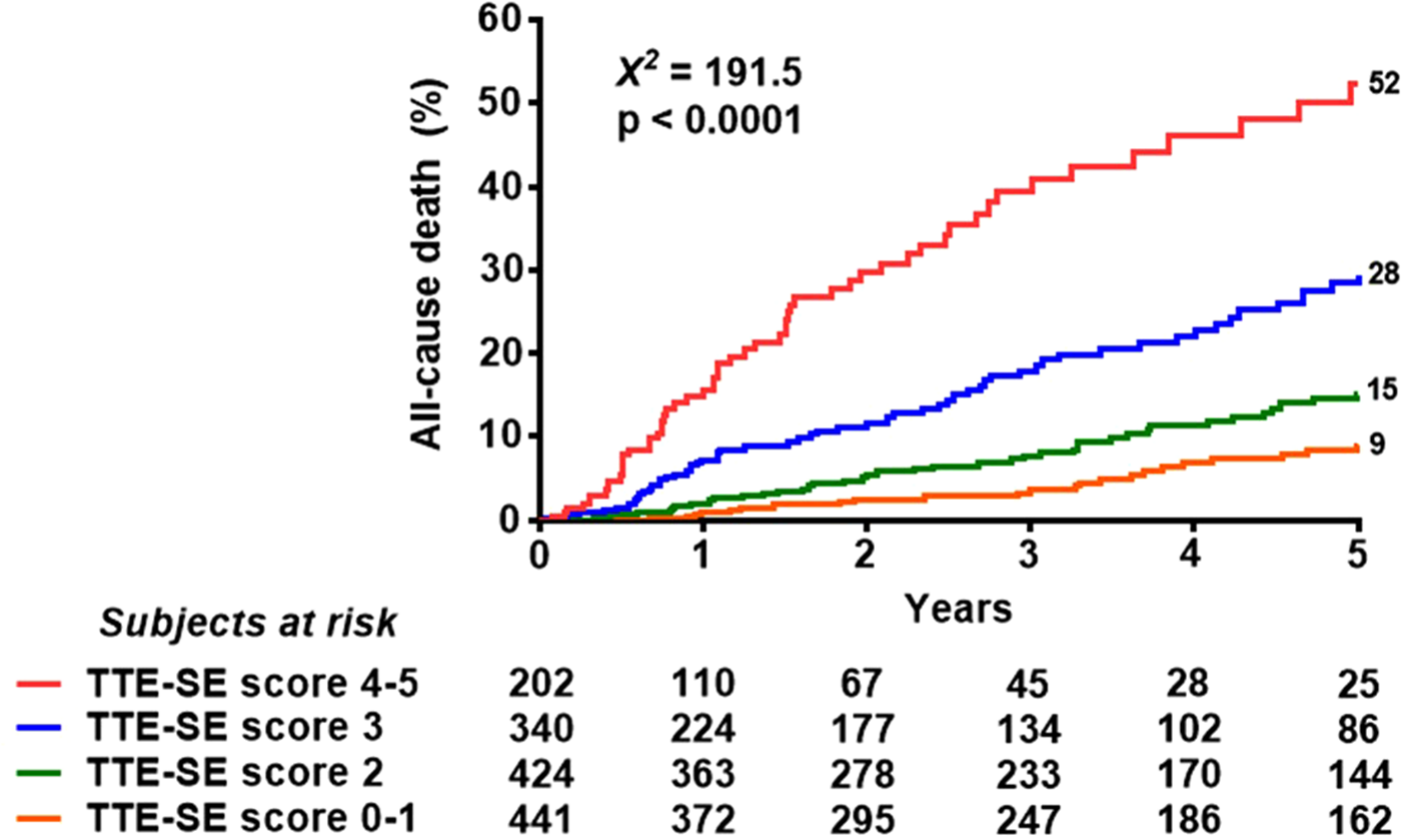
Reverse Kaplan-Meier survival curves. Death rate according to the composite TTE-SE score in the overall population.

Univariable and multivariable prognostic predictors are reported in Table 2. Independent predictors of death were EF (HR: 0.961, 95% CI: 0.945–0.977, *p* < 0.0001), high CFV (HR: 1.748, 95% CI: 1.345–2.270, *p* < 0.001), reduced CFVR (HR: 2.314, 95% CI: 1.685–3.179, *p* < 0.001), stress-induced ischemia (HR: 1.925, 95% CI: 1.404–2.638, *p* < 0.001), and LVCR (HR: 0.507, 95% CI: 0.302–0.851, *p* = 0.010). The annual mortality rate was lowest (1.0%) in patients with a score of 0 (*n* = 61) and highest (31.9%) in patients with a score of 5 (*n* = 15, *p* < 0.001) (Figure 4). Survival progressively worsened with higher score values (Figure 5). In the multivariable analysis, SE score (score 0 = reference value, 1) was an independent predictor of mortality with score = 3 (HR 6.174, 95% CI: 1.952–19.522; *p* = 0.002), score = 4 (HR 12.956, 95% CI: 4.055–41.399; *p* < 0.001), and score = 5 (HR 30.689, 95% CI: 8.113–116.086; *p* < 0.001). By using an interactive stepwise procedure, the global *X*^2^ of the clinical model for mortality was 132.4 (*p* < 0.0001); the inclusion of the TTE-SE score 3–5 increased it to 192.1 (45% increase; *p* < 0.0001).

**Table 2:** Predictors of all-cause mortality.

Variables	Univariable logistic regression analysis		Multivariable logistic regression analysis	
	HR (95% CI)	*p*	HR (95% CI)	*p*
Age (*years*)	1.062 (1.047–1.077)	*<0*.*001*	1.051 (1.037–1.066)	*<0*.*001*
Sex (*male*)	1.203 (0.903–1.603)	*0*.*207*		* *
Hypertensive patients	1.189 (0.913–1.548)	*0*.*198*		* *
Beta-blockers therapy	1.170 (0.912–1.502)	*0*.*217*		* *
Diabetic patients	1.222 (0.950–1.571)	*0*.*118*		* *
Prior MI	1.037 (0.808–1.332)	*0*.*775*		* *
LV EF (%)	0.956 (0.943–0.968)	*<0*.*001*	0.961 (0.946–0.976)	*<0*.*001*
CFV of LAD ≥ 32 cm/s	2.407 (1.871–3.096)	*<0*.*001*	1.748 (1.345–2.270)	*<0*.*001*
CFVR of LAD ≤ 2.0	3.908 (2.903–5.250)	*<0*.*001*	2.314 (1.685–3.179)	*<0*.*001*
LVCR	0.524 (0.324–0.847)	*<0*.*001*	0.507 (0.302–0.851)	*0*.*010*
Stress-induced ischemia	2.223 (1.642–3.009)	*<0*.*001*	1.925 (1.404–2.638)	*<0*.*001*
TTE-SE score = 0	1	* *	1	* *
TTE-SE score = 1	1.516 (0.464–4.952)	*0*.*491*		* *
TTE-SE score = 2	2.968 (0.932–9.444)	*0*.*066*		* *
TTE-SE score = 3	6.174 (1.952–19.552)	*0*.*002*		* *
TTE-SE score = 4	12.958 (4.055–41.399)	*<0*.*001*		* *
TTE-SE score = 5	30.689 (8.113–116.056)	*<0*.*001*		* *

MI, myocardial infarction; LV EF, left ventricular ejection fraction; LVCR, left ventricular contractile reserve; EF, ejection fraction; CVF, coronary flow velocity; LAD, left anterior descending coronary artery; CFVR, coronary flow velocity reserve; TTE -SE, trans-thoracic stress echo.

Since exercise and dobutamine are nominally different from vasodilators (dipyridamole and adenosine) we also separately analyzed the 2 groups. Considering only patients with exercise or dobutamine (*n* = 199, 19, deaths, 9.5%), myocardial ischemia (HR 3.780, 95% CI: 1.264–11.300, *p* = 0.017) and CFVR (HR 3.050, 95% CI: 1.090–8.535, *p* = 0.034) showed the highest value in predicting outcome at univariable analysis. Considering only patients with dipyridamole or adenosine (*n* = 1,209, 234 deaths, 19.4%), CFVR (HR: 4.015. 95% CI: 2.942–5.478, *p* < 0.001), CFV > 32 cm/s (HR: 2.460. 95% CI: 1.880–3.202, *p* < 0.001), and inducible ischemia (HR 2.273, 95% CI: 1.641–3.150, *p* < 0.001) showed the highest value in predicting outcome, followed by resting ejection fraction (HR 0.938, 95% CI: 0.922–0.954, *p* < 0.001) (Central Illustration).

**CENTRAL ILLUSTRATION:**
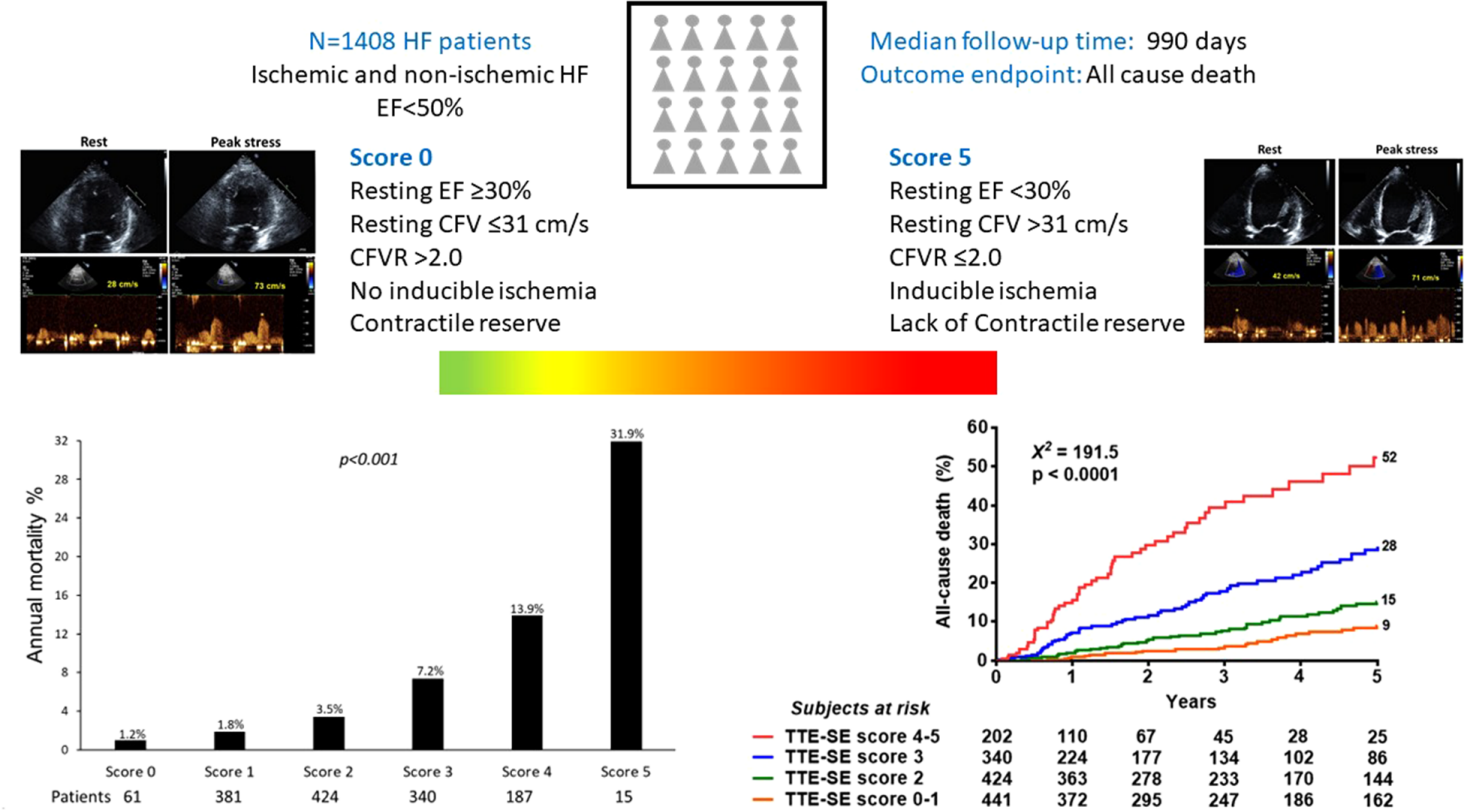
Patients with the same phenotype of HF and reduced/mildly reduced EF of ischemic or nonischemic etiology can be differentiated in different endotypes based on the interaction between resting EF, resting CFV, CFVR, LVCR, and inducible RWMA. The annualized death rate (left lower panel) shows a steep gradient from score 0 (all 5 parameters are normal, annual mortality rate 1.2%) to score 5 (all parameters abnormal, annual mortality rate 31.9%).

## Discussion

In this study, we simultaneously assessed wall motion and coronary flow, at rest and during stress, in patients with HF and reduced/mildly reduced EF, followed for a median of almost 3 years. We demonstrate that: 1- EF, resting CFV, CFVR, LVCR, and inducible RWMA have independent prognostic value; 2- the worse outcome is associated with the combination of severely reduced resting EF, high resting CFV flow, blunted CFVR, absence of LVCR, and inducible RWMA; 3- the stratification obtained with TTE + SE is equally effective in HF of ischemic and non-ischemic origin. TTE + SE is an effective prism to unmask the profound heterogeneity of endotypes and risk hidden behind the same HF phenotype. The underlying etiology (with or without CAD) of DCM was not a significant determinant of prognosis, as shown by lack of significance of underlying condition (CAD vs. non-CAD) at univariable analysis (see Table 2) and similar risk values in the separate analysis of the CAD and no-CAD cohort.

This is especially relevant in a population with heart failure since non-cardiac comorbidities such as diabetes mellitus, anemia, renal disease, and cancer history may all impact the chosen end-point of all-cause death.

## Comparison with previous studies

The data of the present study showing the prognostic value of resting EF, stress-induced RWMA, LVCR, resting CFV, and CFVR are broadly consistent with previous evidence obtained with different techniques in HF patients. EF remains the simplest and most widely used parameter to assess global LV systolic function, with a steep rise in mortality for values <30% in ischemic and non-ischemic patients (11). The prognostic value of EF < 30% is confirmed in the present study, despite the known conceptual limitations of EF due to its dependency on heart rate, loading conditions, LV size (12), and the variability of measurements with 2-dimensional echocardiography (13).

Stress-induced RWMA (synonymous with ischemia in patients with coronary artery disease) is a powerful predictor of death, especially when superimposed on a reduced resting LV function (14, 15). We observed that the adverse impact of inducible RWMA was present in both patients with HF of non-ischemic origin and those with HF of ischemic origin. An anatomic-functional mismatch can be observed in patients with angiographically normal coronary arteries also as a sign of incipient cardiomyopathy, with an abnormal response due to a myocardial or coronary microvascular, rather than coronary macrovascular, etiology. Inducible RWMA with angiographically normal coronary arteries is associated with a worse outcome (16).

LVCR (synonymous with myocardial viability in patients with coronary artery disease) is associated with an improvement in resting LV function, irrespective of treatment, in both ischemic and non-ischemic patients (17–21).

The results of the present study also confirm the powerful prognostic value of CFVR with TTE, shown by single-center (22) and multicenter (23) studies on non-ischemic HF patients, and recently corroborated by a meta-analysis of 9 studies on 7,174 patients which showed a >4-fold increased risk of mortality with abnormal CFVR in all-comers, with or without HF (24). The present study also shows the independent value of resting CFV in predicting survival, as suggested by previous study in populations with chronic coronary syndromes and EF > 50% (25). The value of a reduced CFVR in heart failure was already shown in a previous publication of our study consortium but the present study has important differences from the previous one (23). First, only 192 patients of the previous report were considered, since all patients with preserved ejection fraction (*n* = 270) were excluded from the present analysis, restricted to patients with resting EF < 50%. Second, 923 new patients (with CAD and/or recruited after 2019 and or studied with tests different from vasodilators and/or with inducible regional wall motion abnormality) were added to the present updated analysis. Third, we included in the analysis variables such as resting CFV, inducible ischemia, and myocardial viability excluded from the previous analysis. Fourth, thanks to the larger sample size and extended follow-up all-cause death was the only end-point included in the analysis. We had 253 all-cause deaths in the present series, and only 41 in the previous manuscript (23). Therefore, despite the coherence of results and similar methodology, the present study is substantially different for the previous one for number and type of patients recruited, selection criteria, stress modalities employed, parameters analyzed, outcome measures, and conclusions. An increased resting CFV is associated with a functional alteration of coronary microcirculation, and a reduced CFVR is compatible with a fixed ceiling of the coronary flow for structural coronary microvascular disease (26). The present study is consistent with the available evidence and also unique in several aspects. The current study was prospectively designed on a multicenter basis. We accepted different types of physical and pharmacological stresses. We focused on the relatively homogeneous subset with HF and reduced/mildly reduced EF. We integrated novel markers based on coronary physiology such as resting CFV and CFVR with other established biomarkers of risk based on wall motion, such as EF, LVCR, and RWMA. Due to the large sample size and relatively long follow-up, we evaluated all-cause death as the only significant endpoint.

## Clinical implications

A comprehensive approach is needed to fully characterize the physiological complexity of LV dysfunction in HF. The evidence provided by the present study suggests that the information on EF, LVCR, and inducible ischemia is important for risk stratification, independently of the underlying ischemic or non-ischemic etiology in patients with DCM. It also can be integrated with other relevant information additional to wall motion imaging, simultaneously obtained in the same setting, on resting CFV and CFVR. These parameters can be collected in a one-stop-shop of TTE and SE, in <1-h considering preparation, acquisition, and analysis time, at an affordable cost.

## Study limitations

The study has unavoidable limitations inherent to the observational study design.

Regrettably, the prolonged enrollment period in this study has introduced a significant source of heterogeneity with regard to both follow-up duration and heart failure treatments.

Intercurrent therapy, revascularization procedures, and comorbidities were noted but could not be controlled. Different laboratories used different stress modalities based on their experience and patient's characteristics, and although guidelines recommend exercise for ischemia, dobutamine for viability, and vasodilators for assessing CFVR, it is established that also exercise and dipyridamole may provide accurate information on myocardial viability, dobutamine and dipyridamole are equally powerful ischemic stresses as exercise at appropriately high doses, and the vasodilatory stimulus of exercise and dobutamine is comparable to dipyridamole or adenosine, although technicalities are more challenging and feasibility rate lower with exercise (1, 2, 27). We assessed coronary flow physiology only in LAD coronary artery, but the prognostic value of this index has been abundantly shown in patients with coronary artery disease and in patients with non-ischemic HF. The alterations observed in the LAD are representative of those found in other coronary territories since they are due to the diffuse nature of coronary microvascular involvement (28).

The cut-off point of >32 cm/s for resting CFV was determined by ROC analysis and was identical to the cut-off previously reported for predicting death in a different patient population of patients with chronic coronary syndromes and EF > 50% (25). The actual discrimination was not very robust, but better results are obtained considering CFV as a continuous, not binary, response, since risk increases progressively for highest quintiles (25).

We considered all-cause death as the only endpoint and did not include other disease-specific endpoints such as cardiovascular causes of death. However, all-cause mortality is not affected by bias in classifying the causes of death, can capture unexpected lethal side effects of medical care, and is easily and reliably accessed through a national database (29). This approach allows for ascertaining outcomes of interest with simple interrogation of electronic health records while maintaining the integrity and reliability of study results (30).

## Conclusions

The combination of mildly reduced resting EF, low resting CFV, preserved CFVR, the presence of LV CR, and the absence of inducible RWMA is associated with improved outcomes in both ischemic and non-ischemic HF. This information can be obtained noninvasively with resting TTE and SE, using either pharmacological or exercise stress, with the identification of a spectrum of endotypes associated with profoundly heterogeneous risk and clustered under the same phenotype of HF with reduced/ mildly reduced EF.

## Data Availability

The raw data supporting the conclusions of this article will be made available by the authors, without undue reservation.
