# Supplementary material for: Echocardiographic functional determinants of survival in heart failure with abnormal ejection fraction
Source: Front Cardiovasc Med. 2023 Nov 21;10:1290366. doi: 10.3389/fcvm.2023.1290366 (PMC10699198; doi:10.3389/fcvm.2023.1290366)
Supplement: Supplementary file 1 [file Datasheet1.docx]

**Appendix**

**On behalf of the Stress echo 2030 study group:**

**ARGENTINA**: Cardiodiagnosticos, Investigaciones Medicas, Buenos Aires, Argentina: Jorge Lowenstein ([lowensteinjorge@hotmail.com](mailto:lowensteinjorge@hotmail.com)); Rosina Arbucci ([rosinaarbucci@hotmail.com](mailto:rosinaarbucci@hotmail.com)); Diego M. Lowenstein Haber ([lowediego@hotmail.com](mailto:lowediego@hotmail.com)); Sofia Marconi ([sofi_1151@hotmail.com](mailto:sofi_1151@hotmail.com)); Pablo M Merlo ([pablommerlo@gmail.com](mailto:pablommerlo@gmail.com))

Hospital Echocardiography Laboratory, Ramos Mejia Hospital, Buenos Aires, Argentina: Miguel Amor ([miguelamor68@gmail.com](mailto:miguelamor68@gmail.com)); Hugo Mosto ([hmosto@gmail.com](mailto:hmosto@gmail.com)); Michael Salamé ([michael.f.salame@gmail.com](mailto:michael.f.salame@gmail.com)); Patricia Carral ([patriciabarral06@gmail.com](mailto:patriciabarral06@gmail.com)); Germán Souto ([germansouto87@gmail.com](mailto:germansouto87@gmail.com))

División de Cardiología, Hospital de Clínicas José de San Martín, Buenos Aires, Argentina: Ariel Saad ([arielsaad@gmail.com](mailto:arielsaad@gmail.com))

**BELGIUM:** Department of Cardiology, Antwerp University Hospital, 2650 Edegem, Belgium: Caroline M. Van De Heyning (carovdh@msn.com)

**BOSNIA AND HERZEGOVINA**: Clinic of Cardiovascular Diseases, University of Banja Luka University Clinical Centre of the Republic of Srpska: Tonino Bombardini ([tbombardini@yahoo.it](mailto:tbombardini@yahoo.it)); Tamara Kovačević Preradović ([tamara.kovacevic@medicolaser.info](mailto:tamara.kovacevic@medicolaser.info))

**BRASIL**: Cardiology Division, Hospital San José, Criciuma, Brasil: Clarissa Borguezan-Daros ([clarissabdaros@cardiol.br](mailto:clarissabdaros@cardiol.br))

Hospital de Clinicas UFPR, Medicine Department, Federal University of Paranà, Curitiba, Brasil: Ana Cristina Camarozano (a.camarozano@yahoo.com.br)

**BULGARIA:** Heart and Brain Center of Excellence, University Hospital, Pleven, Bulgaria ([martina_vl@abv.bg](mailto:martina_vl@abv.bg)); Iana Simova (ianasimova@gmail.com)

**CHINA:** Department of Cardiovascular Ultrasound and Non-invasive Cardiology, Sichuan Provincial People's Hospital, China: Zhang Hongmei ([oiczhm@163.com](mailto:oiczhm@163.com)); Yi Wang ([wangyihdl@126.com](https://webmail.cnr.it/webmail/imp/dynamic.php?page=mailbox)); Ding Geqi ([pldgq123@163.com](mailto:pldgq123@163.com))

Key laboratory of ultrasound in cardiac electrophysiology and biomechanics,
The Affiliated Sichuan Provincial People's Hospital of Electronic Science and Technology University of China,
Chengdu, China: Zhang Qingfeng ([qingfengzhang518@126.com](mailto:qingfengzhang518@126.com))

Hebei, China: Yue Heng Wang ([279087481@qq.com](mailto:279087481@qq.com))

**HUNGARY:** Institute of Family Medicine, University of Szeged, Hungary: Albert Varga ([varga.albert@med.u-szeged.hu](mailto:varga.albert@med.u-szeged.hu)); Gergely Agoston (drgergoagoston@gmail.com)

Second Department of Internal medicine and Cardiology Center, University Hospital, Szeged, Hungary, and Elisabeth Hospital, Internal Medicine Department, Hódmezővásárhely, Hungary: Attila Palinkas ([palinkasa@hotmail.com](mailto:palinkasa@hotmail.com)); Robert Sepp ([sepprobert@gmail.com](mailto:sepprobert@gmail.com)); Eszter D. Palinkas (palinkaseszti@hotmail.com)

**ISRAEL**: Chief of Echocardiography Unit, Soroka University Medical Center, Israele: Sergio Kobal ([serkobal@clalit.org.il](mailto:serkobal@clalit.org.il))

**ITALY:** Cardiology Division, Fatebenefratelli Hospital, Benevento, Italy: Quirino Ciampi ([qciampi@gmail.com](mailto:qciampi@gmail.com)); Bruno Villari ([brunovillari@gmail.com](mailto:brunovillari@gmail.com))

Cardiology Department, San Luca Hospital, Lucca, Italy: Lauro Cortigiani ([lacortig@tin.it](mailto:lacortig@tin.it))

PO Umberto I°, Nocera Inferiore (ASL Salerno): Antonello D’Andrea ([antonellodandrea@libero.it](mailto:antonellodandrea@libero.it))

Cardiology Department, Parma University Hospital, Italy: Nicola Gaibazzi ([ngaibazzi@gmail.com](mailto:ngaibazzi@gmail.com)); Domenico Tuttolomondo ([d.tuttolomondo@hotmail.it](mailto:d.tuttolomondo@hotmail.it))

Department of Cardiology, Ospedale per gli Infermi, Faenza, Ravenna, Italy: Elisa Merli ([elisamerli@libero.it](mailto:elisamerli@libero.it))

Cardiothoracic Department, University of Pisa, Italy: Doralisa Morrone ([doralisamorrone@gmail.com](mailto:doralisamorrone@gmail.com))

SOD Diagnostica Cardiovascolare, DAI Cardio-Toraco-Vascolare, e Cardiomyopathy unit, Azienda Ospedaliera-Universitaria Careggi, Italy: Fabio Mori ([morif@aou-careggi.toscana.it](mailto:morif@aou-careggi.toscana.it)); Maria Grazia D’Alfonso ([mariagrazia.dalfonso@gmail.com](mailto:mariagrazia.dalfonso@gmail.com)); Iacopo Olivotto ([iacopo.olivotto@unifi.it](mailto:iacopo.olivotto@unifi.it)); Annamaria Del Franco (annamaria.delfranco@gmail.com);

Cardiology Department and Echocardiography Lab, University Hospital "San Giovanni di Dio e Ruggi d'Aragona," Salerno, Italy: Rodolfo Citro ([rodolfocitro@gmail.com](mailto:rodolfocitro@gmail.com))

Azienda Ospedaliera Rilevanza Nazionale A. Cardarelli Hospital, Naples, Italy: Rosangela Cocchia ([rosangelacocchia@hotmail.com](mailto:rosangelacocchia@hotmail.com)); Eduardo Bossone ([ebossone@hotmail.com](mailto:ebossone@hotmail.com))

Villa Salus Foundation, IRCCS San Camillo Hospital, Venice, Italy: Fausto Rigo ([faustorigo@alice.it](mailto:faustorigo@alice.it))

ASST Santi Paolo e Carlo. Presidio Ospedale San Paolo. Milano: Francesca Bursi ([francescabursi@gmail.com](mailto:francescabursi@gmail.com))

Ospedale San Camillo, Cardiology Division, Rome, Italy: Federica Re ([re.federica77@gmail.com](mailto:re.federica77@gmail.com))

Cardiology Hospital, Policlinico University Hospital of Bari, Italy: Paolo Colonna ([colonna@tiscali.it](mailto:colonna@tiscali.it)); Ilaria Dentamaro ([ilaria.dentamaro@hotmail.it](mailto:ilaria.dentamaro@hotmail.it))

Cardiology Division, San Carlo Hospital, Potenza, Italy: Marco Fabio Costantino ([marcofabiocostantino@tiscali.it](mailto:marcofabiocostantino@tiscali.it))

Ospedale Moscati Avellino, Cardiology Division, : Fiorenzo Manganelli ([fioreman@gmail.com](mailto:fioreman@gmail.com))

**LITHUANIA**: Celutkiene Centre of Cardiology and Angiology, Clinic of Cardiac and Vascular Diseases, Faculty of Medicine, Institute of Clinical Medicine, Vilnius University, LT-03101 Vilnius, Lithuania: Jelena Celutkiene ([Jelena.Celutkiene@santa.lt](mailto:Jelena.Celutkiene@santa.lt))

**MEXICO:** Instituto Nacional de Cardiologia Ignacio Chavez, Mexico City, Mexico: Hugo Rodriguez-Zanella (drzanella@gmail.com)

**POLAND:** Department of Internal Disease and Clinical Pharmacology, Lodz, Poland: Karina Wierzbowska-Drabik ([wierzbowska@ptkardio.pl](mailto:wierzbowska@ptkardio.pl))

Chair of Cardiology, Bieganski Hospital, Medical University, Lodz, Poland: Jaroslaw D. Kasprzak ([wierzbowska@ptkardio.pl](mailto:wierzbowska@ptkardio.pl))

University of Silesia, Cardiology Department, Katowice, Poland: prof. Maciej Haberka maciejhaberka@gmail.com

**RUSSIA:** Cardiology Research Institute, Tomsk National Research Medical Centre of the Russian Academy of Sciences, Tomsk, Russian: Tamara Ryabova ([rtr@cardio-tomsk.ru](mailto:rtr@cardio-tomsk.ru)); Alexander Vrublevsky (avr@cardio-tomsk.ru); Alla Boshchenko (allabosh@mail.ru)

Department of Internal Medicine with a course in Cardiology and functional Diagnostics at the Medical Institute of the Peoples' Friendship University of Russia, Moscow: Ayten Safarova ([aytensaf@mail.ru](mailto:aytensaf@mail.ru)); Tatiana Timofeeva ([timtan@bk.ru](https://webmail.cnr.it/webmail/imp/dynamic.php?page=mailbox))

Cardiology Department, Research Cardiology Center “Medika”, Saint Petersburg, Russian Federation, : Angela Zagatina (zag_angel@yahoo.com)

**SERBIA:** Department of Noninvasive Cardiology, Institute for Cardiovascular Diseases Dedinje, School of Medicine, Belgrade, Serbia: Aleksandra Nikolic ([nikolicdrsasa@gmail.com](mailto:nikolicdrsasa@gmail.com)); Miodrag Ostojic ([mostojic2011@gmail.com](mailto:mostojic2011@gmail.com))

Clinical Cardiology Department, Clinical Hospital Zvezdara, Medical School, University of Belgrade, Serbia: Milica Dekleva ([dekleva.milica@gmail.com](mailto:dekleva.milica@gmail.com))

Cardiology Clinic, University Center Serbia, Medical School, University of Belgrade, Serbia: Ana Djordievic-Dikic ([skali.ana7@gmail.com](mailto:skali.ana7@gmail.com)); Nikola Boskovic ([belkan87@gmail.com](mailto:belkan87@gmail.com)); Vojislav Giga ([voja2011@yahoo.com](mailto:voja2011@yahoo.com)); Milorad Tesic ([misa.tesic@gmail.com](mailto:misa.tesic@gmail.com)); Srdjan Dedic; Branko Beleslin (branko.beleslin@gmail.com)

**SPAIN:** CHUAC- Complexo Hospitalario Universitario A Coruna- University of A Coruna, La Coruna, Spain: Jesus Peteiro Vazquez ([Jesus.Peteiro.Vazquez@sergas.es](mailto:Jesus.Peteiro.Vazquez@sergas.es))

**THAILANDIA**: Division of Cardiology, Department of Medicine, Siriraj Hospital, Mahidol University, Bangkok, Thailand: Nithima Chaowalit Ratanasit ([nithimac@hotmail.com](mailto:nithimac@hotmail.com))

**USA:** Department of Cardiovascular Medicine, Mayo Clinic, Rochester, Minnesota, USA: Patricia A Pellikka ([pellikka.patricia@mayo.edu](mailto:pellikka.patricia@mayo.edu)); Adelaide M. Arruda-Olson ([ArrudaOlson.Adelaide@mayo.edu](mailto:ArrudaOlson.Adelaide@mayo.edu)); Ratnasari Padang ([Padang.Ratnasari@mayo.edu](mailto:Padang.Ratnasari@mayo.edu)); Garvan C. Kane ([kane.garvan@mayo.edu](mailto:kane.garvan@mayo.edu)); Hector R. Villarraga ([Villarraga.Hector@mayo.edu)](mailto:Villarraga.Hector@mayo.edu)%20)

**SIECVI-MAYO core team**: Eugenio Picano (Chairman, eugeniopicanoofficial@yahoo.com); Patricia A Pellikka (Co-chair, pellikka.patricia@mayo.edu); Quirino Ciampi (Principal Investigator, qciampi@gmail.com); Ylenia  Bartolacelli (Paediatric Cardiology and Adult Congenital Heart Disease Unit, S. Orsola-Malpighi Hospital, Bologna, Italy ylenia.bartolacelli@gmail.com), Andrea Barbieri (REDCap for Data Archiving, barbieriandrea65@gmail.com); Giovanni Benfari (University of Verona, Verona, Italy: [giovanni.benfari@gmail.com](mailto:giovanni.benfari@gmail.com)), Mauro Pepi (SIECVI President, Mauro.Pepi@cardiologicomonzino.it); Scipione Carerj (SIECVI President-elect scipione2@interfree.it)
